# A Case Demonstration of the Open Health Natural Language Processing Toolkit From the National COVID-19 Cohort Collaborative and the Researching COVID to Enhance Recovery Programs for a Natural Language Processing System for COVID-19 or Postacute Sequelae of SARS CoV-2 Infection: Algorithm Development and Validation

**DOI:** 10.2196/49997

**Published:** 2024-09-09

**Authors:** Andrew Wen, Liwei Wang, Huan He, Sunyang Fu, Sijia Liu, David A Hanauer, Daniel R Harris, Ramakanth Kavuluru, Rui Zhang, Karthik Natarajan, Nishanth P Pavinkurve, Janos Hajagos, Sritha Rajupet, Veena Lingam, Mary Saltz, Corey Elowsky, Richard A Moffitt, Farrukh M Koraishy, Matvey B Palchuk, Jordan Donovan, Lora Lingrey, Garo Stone-DerHagopian, Robert T Miller, Andrew E Williams, Peter J Leese, Paul I Kovach, Emily R Pfaff, Mikhail Zemmel, Robert D Pates, Nick Guthe, Melissa A Haendel, Christopher G Chute, Hongfang Liu, National COVID Cohort Collaborative, The RECOVER Initiative

**Affiliations:** 1 Department of Artificial Intelligence and Informatics Mayo Clinic Rochester, MN United States; 2 McWilliams School of Biomedical Informatics University of Texas Health Sciences Center at Houston Houston, TX United States; 3 Department of Learning Health Sciences University of Michigan Medical School Ann Arbor, MI United States; 4 Institute for Pharmaceutical Outcomes and Policy College of Pharmacy University of Kentucky Lexington, KY United States; 5 Division of Biomedical Informatics Department of Internal Medicine University of Kentucky Lexington, KY United States; 6 Division of Health Data Science University of Minnesota Medical School Minneapolis, MN United States; 7 Department of Biomedical Informatics Columbia University Irving Medical Center New York, NY United States; 8 Department of Biomedical Informatics Stony Brook Medicine Stony Brook, NY United States; 9 Division of Nephrology Stony Brook Medicine Stony Brook, NY United States; 10 TriNetX LLC Cambridge, MA United States; 11 Clinical and Translational Science Institute Tufts Medical Center Boston, MA United States; 12 Institute for Clinical Research and Health Policy Studies Tufts Medical Center Boston, MA United States; 13 North Carolina Translational and Clinical Sciences Institute University of North Carolina School of Medicine Chapel Hill, NC United States; 14 University of Virginia Charlottesville, VA United States; 15 Department of Population Health New York University Grossman School of Medicine New York, NY United States; 16 University of Colorado Anschutz Medical Campus Denver, CO United States; 17 Schools of Medicine, Public Health, and Nursing Johns Hopkins University Baltimore, MD United States; 18 see Acknowledgments

**Keywords:** natural language processing, clinical information extraction, clinical phenotyping, extract, extraction, NLP, phenotype, phenotyping, narratives, unstructured, PASC, COVID, COVID-19, SARS-CoV-2, OHNLP, Open Health Natural Language Processing

## Abstract

**Background:**

A wealth of clinically relevant information is only obtainable within unstructured clinical narratives, leading to great interest in clinical natural language processing (NLP). While a multitude of approaches to NLP exist, current algorithm development approaches have limitations that can slow the development process. These limitations are exacerbated when the task is emergent, as is the case currently for NLP extraction of signs and symptoms of COVID-19 and postacute sequelae of SARS-CoV-2 infection (PASC).

**Objective:**

This study aims to highlight the current limitations of existing NLP algorithm development approaches that are exacerbated by NLP tasks surrounding emergent clinical concepts and to illustrate our approach to addressing these issues through the use case of developing an NLP system for the signs and symptoms of COVID-19 and PASC.

**Methods:**

We used 2 preexisting studies on PASC as a baseline to determine a set of concepts that should be extracted by NLP. This concept list was then used in conjunction with the Unified Medical Language System to autonomously generate an expanded lexicon to weakly annotate a training set, which was then reviewed by a human expert to generate a fine-tuned NLP algorithm. The annotations from a fully human-annotated test set were then compared with NLP results from the fine-tuned algorithm. The NLP algorithm was then deployed to 10 additional sites that were also running our NLP infrastructure. Of these 10 sites, 5 were used to conduct a federated evaluation of the NLP algorithm.

**Results:**

An NLP algorithm consisting of 12,234 unique normalized text strings corresponding to 2366 unique concepts was developed to extract COVID-19 or PASC signs and symptoms. An unweighted mean dictionary coverage of 77.8% was found for the 5 sites.

**Conclusions:**

The evolutionary and time-critical nature of the PASC NLP task significantly complicates existing approaches to NLP algorithm development. In this work, we present a hybrid approach using the Open Health Natural Language Processing Toolkit aimed at addressing these needs with a dictionary-based weak labeling step that minimizes the need for additional expert annotation while still preserving the fine-tuning capabilities of expert involvement.

## Introduction

The advent of the electronic health record (EHR) and the wealth of longitudinal clinical data contained therein have afforded tremendous opportunities for both clinical research and digital health applications. Fundamental to the feasibility of both of these applications is the availability of clinical information, which, despite the plethora of raw data available in the EHR, can be nontrivial to extract in a computationally accessible format. This is particularly the case for information only accessible within unstructured data, such as clinical narratives, due to the intrinsic nature of human language. The same information can be expressed in many different ways, making the task of algorithmic extraction and standardization for computational semantic interpretation very challenging. Concurrently, however, as much as 80% of clinically relevant information has been found to only be accessible in unstructured form [1]. The need for computationally accessible information extracted from unstructured data has been particularly highlighted with recent research efforts surrounding the ongoing COVID-19 pandemic, particularly with respect to its postacute sequelae (PASC) [2-5]. PASC is defined as ongoing, relapsing, or new symptoms or other health effects occurring after the acute phase of the SARS-CoV-2 infection (ie, present 4 or more weeks after the acute infection). A substantial portion of the information of interest relevant to PASC, for instance, signs and symptoms, is often recorded only within narrative text and is not otherwise found within structured EHR data [6].

One proposed solution to computational extraction of the information within unstructured text is natural language processing (NLP). While a multitude of approaches to clinical NLP currently exist, several existing limitations in these approaches that slow down the development process are magnified by the ongoing and evolving nature of the PASC task. In previous work, we introduced the Open Health Natural Language Processing (OHNLP) Toolkit (OHNLPTK), an NLP framework aiming to provide NLP capabilities at scale in a standards-compliant and consensus-driven manner. In this work, we will highlight current limitations in NLP algorithm development approaches and illustrate our approach to addressing these issues by using PASC as an NLP algorithm development use case for the OHNLPTK.

### NLP-Based Clinical Information Extraction

Fundamentally, many of the current applications for clinical NLP lie in information extraction [7]: specifically, the identification of the presence of certain clinical concepts within a clinical narrative, determination of whether it applies to the patient to which the clinical document in question pertains (eg, identification of positive or negative, subject, and other clinically relevant contextual information), and normalization such that named entities sharing the same semantic meaning but with differing lexical forms are mapped to a consistent, codified, computationally accessible definition.

In the following subsections, we will briefly discuss existing approaches to each of these tasks as well as several resources that can be used to augment each of these tasks as relating to PASC.

### Clinical Named Entity Recognition

Identification of clinical concept mentions within unstructured text is a named entity recognition (NER) task. Broadly, approaches to this problem can be subdivided into 3 subcategories: symbolic [8-12], statistical (including deep neural) [13-17], and some hybrid of the 2 [18-21], alluding to the approach used to identify the boundaries of the entities within the text itself. Symbolic approaches are typically either expert-driven, where symbolic rule sets are handcrafted by clinical domain experts, or dictionary-based, where various clinical ontologies are mined for lexical variants for matching purposes. Statistical systems bypass such knowledge engineering efforts by training a machine learning system to label concept mentions given a collection of annotated text documents with concept mentions manually annotated by domain experts.

Each of these approaches has its own benefits. Due to their nature of being handcrafted by domain experts, expert-driven approaches can achieve extremely high performance and can be easily fine-tuned to meet application-specific needs and correct any observed errors. Conversely, expert-driven systems tend to be limited in scope to specific concepts due to their need for expert knowledge engineering, which can be expensive both temporally and financially. While this is sufficient for many applications, such an approach is only suitable if sufficient resources are present for the domain experts and the set of concepts that are needed is known. Dictionary-based symbolic systems aim to address this issue. The solution to the domain expertise problem has been through the use of general clinical ontologies and similar vocabulary resources, such as the National Library of Medicine’s Unified Medical Language System (UMLS), either as a basis to construct general dictionaries that cover concepts from a much greater breadth of the clinical domain, although without the manual curation that is afforded to expert-driven systems, or to derive a larger set of lexical variants for a specific set of concepts without the need to engage domain expertise. While these systems tend to not perform as well as expertly curated rule-based systems, generally high performance has been shown to be achievable. For statistical systems, the creation of a high-quality annotated corpus is also an expensive and laborious process. The situation becomes more complicated in cases of multisite collaboration as clinical narratives may contain patient identifier information, making data sharing challenging, and at the same time, the local site may not have the necessary resources for creating annotated corpora. Additionally, they are difficult to fine-tune as there is very little control short of additional annotation and training data manipulation to correct any errors. Models used for statistical approaches include conditional random fields [14,21-23], hidden Markov models [24-26], Bidirectional Encoder Representations from Transformers (BERT) (after finetuning specifically to accomplish the NER task) and BERT-like models (which are particularly prevalent in, but not exclusive to, multilingual use cases) [13,15,27-30], and other neural methods such as recurrent neural networks and convolutional neural networks [17,31-34].

### Contextual Feature Detection for Clinical Named Entities

Unlike in the general domain, certain contextual features pose a great impact on the relevance and meaning of extracted concepts in the clinical domain. Of particular note is a concept’s assertion (asserted vs possible vs hypothetical), negation, temporality, and whether or not it relates to the patient (as opposed to, eg, a family member), as all of these drastically change the relevance of the concept for downstream applications.

Much like for NER, both symbolic and statistical approaches exist for context detection, with many of the same benefits and drawbacks for each. Among the symbolic systems [35-38], the ConText algorithm proposed by Chapman et al [39] is a symbolic system that is widely adopted among clinical NLP implementations. Various statistical approaches have also been proposed, ranging from traditional statistical machine learning methods such as linear kernel support vector machines [40-42] to deep neural methods [43-45]. There is no clear evidence that statistical approaches outperform the widely adopted ConText algorithm [42].

### Concept Normalization Approaches and Available Resources

Identification of named entities and whether they apply to the patient is only part of the problem for clinical information extraction tasks: to be computationally accessible, these named entities must first be mapped to some known coding system such that named entities with differing lexical variations but with the same semantic meaning are grouped in some computationally accessible manner; that is, a computationally accessible thesaurus for the extracted named entities must be constructed.

There are several approaches to this concept normalization (also often referred to as entity linking) problem. One of the side benefits of symbolic NER methods is that they will often have this normalization built in, whether as part of the construction process by domain experts for expert-based systems or due to the nature of their lexical variants being derived from structured ontologies that themselves often act as pseudothesauri, as is the case for the UMLS for dictionary-based systems [10,11,21].

The same is not always true for NER approaches based on statistical methods: while some systems, particularly those trained to extract a specific set of clinical concepts, do incorporate normalization by the very nature of their training approach, other systems trained to perform general named entity recognition do not incorporate such an element. A secondary step must then be taken to perform such a normalization, oftentimes again leveraging ontologies by doing similarity matches against ontology entries [21]. Despite this, it is worthwhile to note that normalization performance is typically inferior to that of symbolic approaches.

Irrespective of the approach, a common theme in clinical NLP is that the extracted named entities are typically mapped to some ontology for later ease of computational access, particularly the UMLS due to its breadth of source vocabularies.

### PASC and the Emergent Phenotyping Workflow Problem

It is worthwhile to note that NLP-derived data often serves as supplemental information, in that it is used to supply information that, while needed for a particular use case, cannot be found in structured data. This is especially true for phenotyping and similar cohort identification tasks such as clinical trial recruitment, which often have specific inclusion and exclusion criteria that draw from information elements in both structured and unstructured data.

When the inclusion or exclusion criteria and features of interest pertain to emergent entities of interest, however, as was the case with COVID-19, existing approaches to constructing NLP algorithms break down. Statistical methods for NER require data to train, which may not yet exist in sufficiently large volumes due to the fact that the entities of interest themselves are emergent. Additionally, it is worthwhile to note that in certain circumstances surrounding emergent clinical entities, drastic changes to clinical workflow and, by extension, the contents of the documentation itself can occur, as was the case with COVID-19.

Symbolic methods, however, may not necessarily fare better. Ontology-derived dictionary-based approaches can fail in these circumstances due to the fact that ontologies and similar resources used may not yet be updated to contain these emergent entities (or may not yet have new or updated names to refer to existing entities as the terminology used changes over time), and their slow update frequency (biyearly, in the case of the UMLS) results in them being unsuitable for dealing with emergent needs. Expert-driven systems, on the other hand, fare better due to their relative ease of fine-tuning, but the limitations faced by expert-based NER still apply, rendering a purely expert-driven solution infeasible for many use cases.

It is important to note that these problems do not only occur with the introduction of emergent diseases, as was the case with the COVID-19 and PASC studies; rather, attempts to construct NLP systems to address these use cases magnify existing limitations that typically only slow down the development process.

Irrespective of whether these limitations merely slow down or completely hinder NLP development for a particular use case, such limitations are undesirable given the increasing demand for NLP to fulfill a variety of information needs. It thus becomes evident that an approach capable of prototyping and developing NLP systems in a more rapid manner is needed that can combine the fine-grained control and rapid prototyping ability of expert-driven systems with the general applicability afforded by dictionary systems. It is this need that motivates the work presented here.

### Usage of NLP for PASC-Related Tasks

With regard to NLP usage in the context of PASC, rather than a focus on NLP algorithm development, NLP has primarily been used indirectly for other tasks within the PASC context. For instance, Bhavnani et al [46] extracted 20 signs or symptoms defined by the CDC as being PASC-related from clinical narratives to identify symptom-based phenotypes for patients with PASC, while Zhu et al [47] fine-tuned various BERT-based models to classify documents pertaining to patients with PASC signs or symptoms. More applicable to this work is work primarily focused on identifying what specific NLP-derived signs or symptoms are appropriate for inclusion in a PASC signs or symptoms extraction task. For instance, Wang et al [48] used MTerms [49] to mine existing UMLS concepts from clinical narratives associated with COVID-19 positivity to build a lexicon of 355 long COVID-19 symptoms. We use their developed lexicon as one of the bases for further development in this study.

### Clinical NLP to Empower Clinical Research and Translation

**Figure 1:**
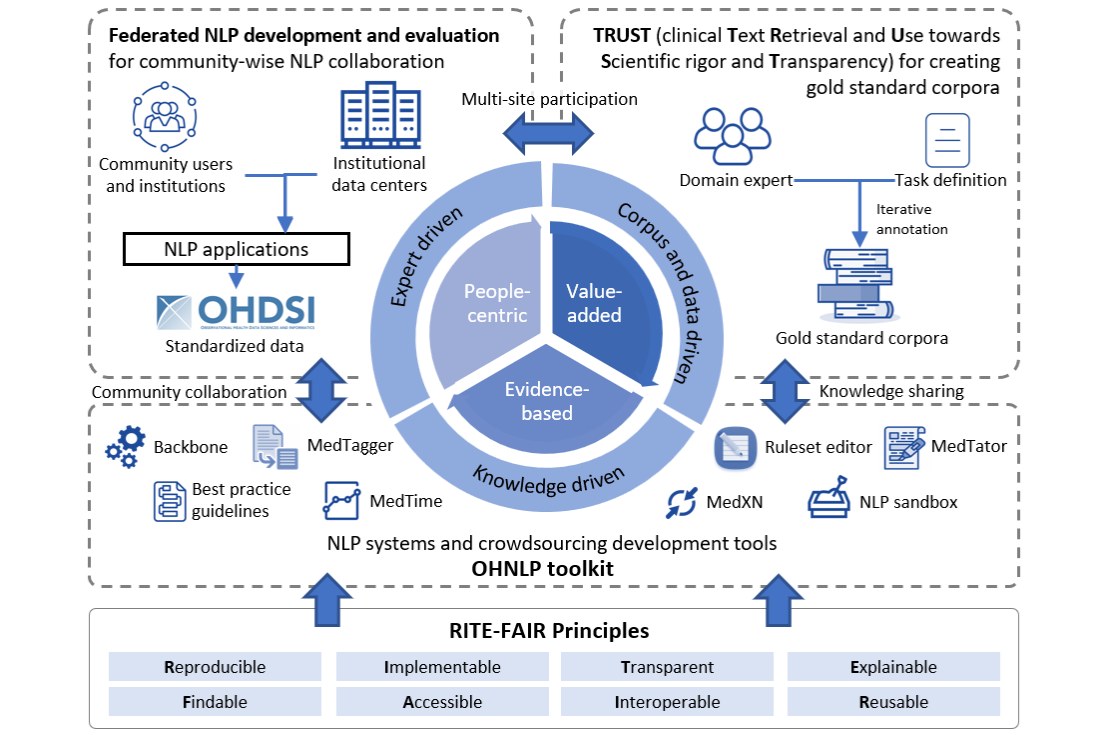
An overview of the Open Health Natural Language Processing ecosystem. FAIR: findable, accessible, interoperable, and explainable; NLP: natural language processing; OHNLP: Open Health Natural Language Processing; RITE: reproducible, implementable, transparent, and explainable.

We developed the OHNLPTK as part of previous work to enable the rapid development and dissemination of NLP algorithms for empowering clinical research and translation [50]. We follow the RITE-FAIR (reproducible, implementable, transparent, explainable-findable, accessible, interoperable, and reusable) principles [51] to ensure scientific rigor and fairness for resources developed, demonstrated, and disseminated for clinical NLP for health. We have released the OHNLPTK to encourage collaboration across the clinical NLP community to address real-world data problems. The toolkit consists of the following components: (1) a federated NLP deployment framework for privacy-preserving clinical NLP enabled by clinical common data models, (2) a clinical text retrieval and use process toward scientific rigor and transparent (TRUST) process, and (3) an open science collaboration toward real-world clinical NLP (Figure 1).

## Methods

### Overview

**Figure 2:**
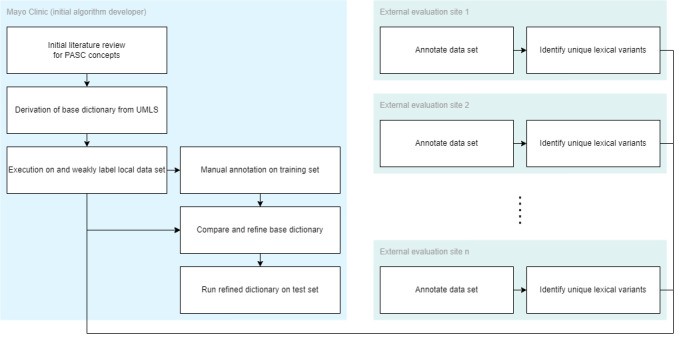
An overview of dictionary construction and federated refinement workflows. PASC: postacute sequelae of SARS CoV-2 infection; UMLS: Unified Medical Language System.

In this study, we report on the use of the OHNLPTK to rapidly develop and prototype an NLP system using a case study on PASC signs and symptoms based on PASC resources available in 2 studies. In the ensuing subsections, we will detail each of the steps associated with the creation and evaluation of the final NLP algorithm. For an overview of the entire process, please refer to Figure 2.

### PASC Resources

As PASC has been recognized nationally, some useful language resources have been developed. Deer et al [52] identified 303 articles published before April 29, 2021, curated 59 relevant manuscripts that described clinical manifestations in 81 cohorts of individuals 3 weeks or more following acute COVID-19, and mapped 287 unique clinical findings to Human Phenotype Ontology (HPO) terms. Wang et al [48] identified 355 long COVID-19 symptoms corresponding to 1520 UMLS concepts, which in turn resulted in 16,466 synonyms identified from 328,879 clinical notes of 26,117 COVID-19 positive patients from their postacute infection period (days 51-110 from the first positive COVID-19 test).

### Dictionary Construction

An initial phenotype definition consisting of textual descriptions, *International Classification of Diseases*, and HPO codes corresponding to clinical entities of interest to PASC was obtained from the aforementioned PASC resources. These entities, where possible, were cross-referenced against the UMLS 2021AB version to obtain an algorithmically derived base dictionary for further refinement. Entities that could not be cross-referenced for enhancement were noted for manual annotation in our second step.

### Dictionary Enhancement and Rule Creation

We collected 128 clinical notes from the post–COVID-19 care clinic at Mayo Clinic and split them into a training set (98 notes) and a testing set (30 notes). For the training set, the algorithmically derived base dictionary was run on the document set to generate a weakly labeled data set that was then loaded into the MedTator web annotation tool [53] for manual review and dictionary refinement. Deficiencies (ie, mentions and concepts that were not identified or contained within the algorithmically defined base dictionary) were identified by domain expert review on the training set. Identified missed lexical variants were then manually linked to an appropriate concept ID and added to the base dictionary. Additionally, any concepts missed during base dictionary generation (ie, a clinical concept that was identified as PASC-relevant in the training set but was not part of those concepts identified in the 2 PASC resources used as a baseline) were manually added. To capture complex contextual information, additional rules were created. For example, for “learning difficulty,” a rule was created to combine a set of terms meaning difficulty with learning, that is, “(%reDIFFICULTY) to (learn, retain, and gain) new information.” The testing set was manually annotated by a single human annotator blindly using the NLP system.

### Concept Normalization

Due to the fact that the base dictionary was derived from a structured metathesaurus, the UMLS concept normalizations to UMLS concept unique identifiers (CUIs) were already present. To be compatible with standardization and cross-compatibility, we opted to further map the UMLS CUIs to associated Observational Health Data Sciences and Informatics (OHDSI) Athena concept IDs [54], which are used as part of the OHDSI Observational Medical Outcomes Partnership (OMOP) common data model. This was done algorithmically using text string matching for concept names, followed by manual linking when an exact text match could not be found. For PASC terms that could not be mapped, we manually reviewed and aligned them with the closest Athena concept identifiers, if available, or mapped them to the HPO identifiers. Concept IDs that could not be mapped to either an Athena concept ID or an HPO identifier (due to emergent entities not being yet present in the source vocabularies) were encoded with custom IDs, and these IDs were recorded for later updates once associated ontologies were updated.

### Lift Analysis and Local Evaluation

Dictionary coverage was compared before and after refinement to evaluate the effect of our weakly supervised refinement approach, specifically with respect to the number of relevant mentions missed by an ontology-based dictionary-only approach.

Beyond being used in the multisite evaluation phase described later in this study, Mayo Clinic’s 30-note testing set was also separately compared against the results from the refined NLP algorithm for in-depth evaluation of NLP and manual annotation mismatches.

### Multisite Evaluation

The resulting NLP algorithm was distributed to 5 sites (University of Minnesota, University of Kentucky, University of Michigan, Stony Brook Medicine, and Columbia University) for evaluation to evaluate the federated evaluation component of the OHNLPTK. Each site manually annotated 10 notes for long COVID-19 signs or symptoms using 2 annotators with an independent adjudicator for disagreements. The resulting text annotations (medical concepts that do not contain PHI) were then returned to the Mayo Clinic for dictionary coverage analysis through comparison against the NLP algorithm.

### Ethical Considerations

Human subject ethics review and informed consent were handled through the individual institution review boards of participating institutions, which gave final approval for this research on the participating patient cohort. The N3C data transfer to National Center for Advancing Translational Sciences is performed under a Johns Hopkins University Reliance Protocol (IRB00249128) or individual site agreements with the National Institutes of Health. All data transmitted to the coordinating site was either manually deidentified or did not contain PII by definition before transmission. No specific compensation was provided to participants as part of this research.

## Results

### Dictionary Construction, Lift Analysis, and Local Evaluation

The final dictionary created from previous PASC resources consists of 12,145 unique text strings, mapped to 2343 HPO or OMOP concept identifiers. Within the Mayo Clinic training or development set, the baseline system detected 8090 PASC concept mentions. After manual verification, we identified that 338 PASC concept mentions were missed, rendering the total number of annotated mentions within the training set to be 8428. The final refined dictionary includes 12,234 unique text strings, mapped to 2366 HPO or OMOP concept identifiers (ie, 23 additional concepts and 89 additional text strings were added). A total of 4 PASC concepts present in the UMLS were captured that could be mapped to neither the OMOP vocabulary nor the HPO (eg, teeth chatter and unrefreshed sleep).

For the local evaluation portion of this study, only the PASC sign and symptom sections of the 30 Mayo Clinic test set notes were compared. In the following report of the results, to facilitate reader understanding, we will use the traditional true positive, false positive, or false negative terminology common to NLP evaluations, despite them not being fully applicable due to varying definitions of what is a true positive depending on what the resulting NLP artifacts are being used for or how PASC-related should be defined for the use case (eg, is it PASC-related if the patient had severe COVID-19 in the past or only if it is explicitly written out as “...consequent to previous COVID-19 infection?”). For more details on this, please refer to the “Discussion” section “On Gaps in the NLP Clinical Information Extraction Subtask.” A total of 1560 annotations were produced by the NLP algorithm, while manual annotation produced 1067 annotations. Of these, 1061 (236 unique text strings ignoring capitalization) would be considered true positives in a traditional NLP evaluation, 489 (445 unique text strings ignoring capitalization) false positives, and 6 (4 unique text strings ignoring capitalization) false negatives. It is important, however, to note that due to certain features of this task, a traditional NLP evaluation is not necessarily fully accurate. For instance, among a substantial portion of the “false positives,” the NLP algorithm made accurate extraction of a sign or symptom that is PASC-related, but human annotation did not occur as the sign or symptom is preexisting and not resulting due to acute COVID-19 infection. For example, the patient has a previous medical history of some of those signs or symptoms, like a headache or migraine, before COVID-19. Similarly, false negatives may not necessarily be attributed to issues with the NLP algorithm. For instance, of the 4 unique text strings composing the false negatives (sexsomnia, taste or smell changes, burning mouth, and sensitivities to noise and light), one (sexsomnia) was not recorded as a PASC-related concept in either of the source articles from which the NLP concepts to extract were defined, but was suggested to be PASC-related within the textual narrative itself and was therefore annotated as such by the human annotator. The other 3 were either caused by a span mismatch or were lexical variants that were neither in the ontologies used to generate the baseline dictionary nor in the training set for manual expert refinement.

### Multisite Evaluation

In Table 1, we present the results from manual annotation after adjudication as well as the dictionary coverage for these annotations.

**Table 1:** Dictionary coverage statistics.

Results	Site 1	Site 2	Site 3	Site 4	Site 5
Number of annotations, n	126	23	171	118	73
Number in dictionary, n	77	20	138	84	65
Coverage ratio, n/n (%)	77/126 (61.1)	20/23 (87)	138/171 (80.7)	84/118 (71.2)	65/73 (89)

In Table 2, we present an analysis of the annotations not covered by the NLP algorithm. Here, we define a new concept as an annotation that was not included as a concept in the original dictionary or rule set, a new variant as an annotation that is an additional lexical variant of a concept existing in the original dictionary or rule set, and an annotation error as an annotation that falls outside of our task definition: for example, COVID-19 or long COVID-19 is not a sign or symptom of COVID-19 or PASC. For a detailed listing of missed terms, please refer to Multimedia Appendix 1.

**Table 2:** Statistics of annotations not covered by dictionary.

Results	Site 1	Site 2	Site 3	Site 4	Site 5
Number of annotations not covered by natural language processing, n	49	3	33	34	8
New concept, n/n (%)	23/49 (46.9)	2/3 (66.7)	9/33 (27.3)	4/34 (11.8)	1/8 (12.5)
New variant, n/n (%)	26/49 (53.1)	1/3 (33.3)	17/33 (51.5)	26/34 (76.5)	4/8 (50)
Annotation error, n/n (%)	0 (0)	0 (0)	7/33 (21.2)	4/34 (11.8)	3/8 (37.5)

## Discussion

### Overview

The COVID-19 pandemic and the associated PASC problem highlighted the importance of having a framework for NLP development that is sufficiently flexible to both provide general concept detection capabilities without requiring extensive domain expertise to craft rule sets or annotate extensive data sets, but also with sufficient flexibility for fine-tuning and addition of concepts that are not present in the base ontologies from which the dictionaries are derived. Additionally, it has highlighted the need for such an NLP development process to be agile in iteration, as the rapidly changing concepts and definitions associated with these emergent concepts are inherently incompatible with the relatively slow process associated with traditional NLP or information extraction (IE) algorithm development (as by the time the algorithm is developed, the definition will have changed). Here, we have presented an approach to NLP algorithm development that allows us to achieve reasonable results on limited data sets with a rapid turnaround time (the entire process was accomplished over a month) by leveraging federated algorithm development and common infrastructure. The resulting NLP algorithm has been published as part of the open-sourced MedTagger NLP framework [9] and is being executed at 10 academic medical centers as part of their NLP data submissions to the National COVID-19 Cohort Collaborative (N3C) data set [55]. In this section, we will first discuss several gaps in the current NLP development process that were suspected and the degree to which the OHNLPTK was able to address them, and, finally, we will discuss several of this study’s limitations and key takeaways.

### On Gaps in the NLP Development Process

Our execution of the PASC use case using the OHNLPTK confirmed many of the suspected gaps in the current NLP ecosystem pertaining to emergent diseases.

First, the potential coverage gap of a purely ontology-derived dictionary-based approach to NLP is highlighted: of the 8428 PASC-related concept mentions within our corpus, the baseline ontology-derived dictionary only identified 8090 mentions, missing 338. These 338 mentions span 167 concepts, highlighting the need for secondary expert-based refinement.

Additionally, this case study highlighted the infeasibility of a purely expert-based approach: there were 1018 unique lexical variants covering PASC-related clinical concepts within our training corpus after dictionary-based weak labeling and expert enhancement. Based on this lexicon size, an expert-based approach to implementing an acceptable NLP system would either be prohibitively expensive resource-wise (by horizontally scaling through adding additional experts) or unreasonably time-consuming, to the point of rendering the approach completely infeasible depending on the study’s time constraints.

While it is difficult to draw conclusions about the viability of statistical approaches based solely on what was done in this case study, it is worthwhile to note that of the 8428 PASC mentions in our training corpus, the vast majority were weakly labeled through dictionary lookups, while the remainder were manually derived through an expert review. It is very likely that the manual effort required to fully annotate the train data set for a fully supervised approach would have been prohibitively expensive for many studies, given these statistics. Additionally, as these 8428 PASC mentions are split in turn across many concepts, there are very few unique mentions or lexical examples per concept. By extension, there is an insufficient number of examples to support training high-performing models for all concepts, and annotation of further training data would be required. In fact, this data problem is further exacerbated.

It is thus evident that a hybrid approach that integrates each of the three approaches of statistical, symbolic expert-driven, and symbolic dictionary-based would be ideal. Based on the results of the execution of our PASC use case, we believe that we have demonstrated that expert refinement of a rule set on top of a data set already weakly labeled by an ontology-derived dictionary is one such viable hybrid approach.

With our use case demonstration, we also show that the OHNLPTK can be leveraged to execute an NLP development process following this approach. An initial dictionary derived from the UMLS can be directly loaded into deployed instances of the OHNLPTK without any additional software modification. Weakly labeled NLP output can be directly piped to OHNLPTK’s MedTator component for expert review, and additional finetuning is autonomously translated into refinement rules that can, in turn, be executed on top of the base dictionary.

### On Gaps in the NLP Clinical Information Extraction Subtask

In this case study, we have strictly focused on clinical information extraction, which has thus far been the core focus for a significant portion of clinical NLP applications. It is important, however, to note that even with a clinical information extraction algorithm, the output may not be wholly applicable and/or useful to the use case. There were multiple instances where our NLP system correctly extracted mentions of some PASC-related terms that were marked as false negatives upon comparison against the gold standard.

Upon further investigation, we discovered that the NLP system correctly identified mentions of the entities in question, but they were not annotated by the annotator as they were not specific to PASC. For instance, while headaches are a valid symptom that is associated with PASC, they may also occur independently due to unrelated reasons. While human annotators have the capability to make this distinction, our NLP system cannot currently make such a distinction and simply naively extracts all valid mentions. Strictly speaking, from the perspective of the NLP-based named entity recognition and linkage subtask (ie, “identify mentions of headaches, and annotate them as such”), the algorithm output is correct. Conversely, however, for real-world use cases such as the PASC investigation use case presented here, not making such a distinction has a significant impact on the practical usability of the NLP artifacts produced. While such contextual differentiation would fall under a different subtask and is not strictly information extraction, the ability to perform this differentiation is crucial for many use cases and is an existing gap in current clinical NLP offerings. We aim to further explore approaches to this differentiation task as part of our future work.

### On Federated Evaluation and Associated Benefits

Before federated evaluation can be done, a common working definition or annotation guideline must be defined. The addition of other data and sites, however, also introduces its own complexity, specifically with respect to the aforementioned issue of how to define a concept mentioned as being “PASC-related,” on which sites had a very broad spectrum of definitions. We concluded as a group that differentiating whether a mention of, for example, a headache is PASC-related ought to be considered a separate task from the clinical information extraction task and that such filtering, if needed, would be done as a separate, post-IE step.

We opted to evaluate the dictionary-based NLP system using dictionary coverage as opposed to a more traditional precision, recall, and *F*_1_-score. This was done because we wished to evaluate the ability of a generic NLP algorithm to meet the needs of multiple institutions in a cost-effective manner. Due to the recency and evolutionary nature of long COVID-19, it follows that each site’s definition (or at least those of the participating annotators) of “PASC or long COVID-19” may differ, thus rendering it difficult to construct a gold standard with a consistent set of concepts. Instead, by allowing individual sites to determine what constitutes a long COVID-19 for their needs, we can evaluate the coverage—that is, to what extent said need is met by the NLP algorithm.

While the initial Mayo-developed NLP system performs reasonably well across the participating sites that returned evaluation results, not all information needs are met in terms of dictionary coverage. We note that site 1 only had 61.1% (77/126) dictionary coverage; of those annotations not covered, 46.9% (23/49) were new concepts. Manual review indicated some of these new concepts were lung-related (pneumothorax, hydropneumothorax, and ground glass attenuation), which suggests site 1’s PASC-related symptoms are slightly different than other sites. A total of 9 of these new concepts (9/23, 39.1%) were the result of a very detailed note (32,000 characters in length) of a complex PASC case where other poor-health and pain-related concepts (gallstones and cholelithiasis) were intermixed with chest pain, lung pain, and difficulty breathing. For all sites, more than a third of the missing annotations were categorized as missing lexical variations of included concepts that did not appear within either the autonomously generated ontology-sourced dictionary or the Mayo documentation set used for dictionary refinement.

Such results illustrate the importance of multisite NLP development, particularly in the latter case, which is one of the pitfalls that would be commonly found in traditional NLP tasks. Our results therefore fundamentally demonstrate the advantage of the high degree of portability resulting from a common NLP infrastructure when discussing generalizable NLP solutions. As gaps in data coverage are assessed across a variety of health care institutions, NLP algorithm refinement is vastly simplified, thus granting greater confidence in the wide-range generalizability of the final solution. Similarly, the addition of these additional sites with their own respective annotators also helps mitigate potential bias associated with single-site or single-annotator definitions of the PASC sign or symptom extraction task.

Beyond the issue of what clinical concepts to include is another issue less addressed in this study: a definition of what sorts of data to include. This is a challenge exacerbated by the fact that, much like how emergent diseases will constantly have an evolving associated concept set, the way they are documented (and their extent) is also constantly changing and subject to high levels of disagreement. This phenomenon can be seen in the wildly different annotated concept counts within the evaluation sites despite sharing the same concept set to annotate: certain sites only considered a specific section of a clinical note explicitly dedicated to documented PASC complications to be appropriate as input data, while others used the entire clinical note for any patient that had visited their long COVID-19 clinic. This wide variance in data inclusion definition further supports the need for federated development and evaluation efforts as outlined in this study to further expose the developed algorithm to this wide variety of data types. Furthermore, the fact that the condition is emergent inherently limits data set sizes; at the time this study was conducted, long COVID-19 clinics were still a relatively novel concept in their initial stages of implementation. A key benefit of such a federated evaluation and iterative refinement process would be to help mitigate the limited amount of data available inherently associated with an emergent condition by spreading out the data set to multiple sources and making it available for the development of a wider variety of documentation types.

### Limitations

Several limitations exist in this study. First, we compared dictionary coverage as an evaluation, rather than doing a traditional NLP system evaluation. This is primarily due to the lack of a fully annotated gold standard to evaluate against, driven primarily by the ongoing evolutionary nature of the pandemic, causing associated documentation to continuously change. This renders a truly scientifically rigorous gold standard difficult to construct, as annotation guidelines must be constantly updated and there will be very little consensus due to a lack of clear clinical guidelines. Instead, we note that this limitation highlights the need for iterative development, which further emphasizes the need for an agile NLP development, evaluation, and refinement process such as the one we present here.

Additionally, one of the emphases of the OHNLP consortium is the organization of multi-institutional, federated evaluation for NLP algorithm development, enabled by a common NLP system deployment (the OHNLPTK). As such, we are currently in the process of disseminating the algorithm presented here to multiple member sites, who will all conduct a formal evaluation of this algorithm.

Finally, it was previously noted that in order to codify and normalize concepts that do not yet exist in controlled ontologies, we introduced our own coding scheme for several concepts. It is important for standardization’s sake, however, to loop back to the original ontologies used and have these concepts incorporated into these source ontologies. This process is ongoing as of the writing of this article.

### Conclusion

The PASC NLP problem has highlighted many of the limitations present with current NLP development approaches. The evolutionary and time-critical nature of the PASC NLP task exacerbates many of these limitations, which previously only presented a slowdown of the development process, into limitations that cause many approaches to be outright infeasible. The need for agile and iterative NLP development is thus made evident. Fundamentally, this can be observed as an amalgamation of wanting the benefits of expert-driven systems while minimizing the time and resource expenditure of expert involvement. Here we have presented a hybrid approach that we believe presents such benefits, with a dictionary-based weak labeling step minimizing the need for additional expert annotation while still preserving the fine-tuning capabilities of expert involvement.

## Appendix

Multimedia Appendix 1 A list of terms not covered by natural language processing (NLP) algorithms.

## Abbreviations

BERT: Bidirectional Encoder Representations from Transformers
CUI: concept unique identifier
EHR: electronic health record
HPO: Human Phenotype Ontology
IE: information extraction
N3C: National COVID-19 Cohort Collaborative
NER: named entity recognition
NLP: natural language processing
OHDSI: Observational Health Data Sciences and Informatics
OHNLP: Open Health Natural Language Processing
OHNLPTK: Open Health Natural Language Processing Toolkit
OMOP: Observational Medical Outcomes Partnership
PASC: postacute sequelae of SARS-CoV-2 infection
RECOVER: Researching COVID to Enhance Recovery
RITE-FAIR: Reproducible, Implementable, Transparent, Explainable - Findable, Accessible, Interoperable, and Reusable
TRUST: text retrieval and use process toward scientific rigor and transparent
UMLS: Unified Medical Language System
