# Supplementary material for: A Case Demonstration of the Open Health Natural Language Processing Toolkit From the National COVID-19 Cohort Collaborative and the Researching COVID to Enhance Recovery Programs for a Natural Language Processing System for COVID-19 or Postacute Sequelae of SARS CoV-2 Infection: Algorithm Development and Validation
Source: JMIR Med Inform. 2024 Sep 9;12:e49997. doi: 10.2196/49997 (PMC11420592; doi:10.2196/49997)
Supplement: Multimedia Appendix 1 [file medinform_v12i1e49997_app1.docx]

**Table S1**.

| Site | Annotation | Frequency | New concept | New variant | Annotation error |
| --- | --- | --- | --- | --- | --- |
| Site 1 | abdominal complaints | 1 |  | Y |  |
| Site 1 | acute deep vein thrombosis | 1 |  | Y |  |
| Site 1 | acute kidney injury | 1 | Y |  |  |
| Site 1 | afib | 1 | Y |  |  |
| Site 1 | AKI | 2 | Y |  |  |
| Site 1 | AOCD | 1 | Y |  |  |
| Site 1 | ARDS | 2 |  | Y |  |
| Site 1 | cholelithiasis | 3 | Y |  |  |
| Site 1 | CKD | 1 | Y |  |  |
| Site 1 | coarse breath sounds | 1 |  | Y |  |
| Site 1 | cortical infarct | 1 |  | Y |  |
| Site 1 | crackles | 2 |  | Y |  |
| Site 1 | deconditioned | 1 |  | Y |  |
| Site 1 | deconditioning | 2 |  | Y |  |
| Site 1 | DOE | 1 |  | Y |  |
| Site 1 | gallstones | 1 | Y |  |  |
| Site 1 | groundglass attenuation | 2 | Y |  |  |
| Site 1 | hydropneumothorax | 3 | Y |  |  |
| Site 1 | hyperbilirubinemia | 1 | Y |  |  |
| Site 1 | hypercalcemia | 2 | Y |  |  |
| Site 1 | hypercoagulability | 1 |  | Y |  |
| Site 1 | hyperkalemia | 2 | Y |  |  |
| Site 1 | hypernatremia | 1 | Y |  |  |
| Site 1 | hypoalbuminemia | 1 | Y |  |  |
| Site 1 | hypokalemia | 1 | Y |  |  |
| Site 1 | hypomagnesemia | 2 | Y |  |  |
| Site 1 | hyponatremia | 2 | Y |  |  |
| Site 1 | immobility | 1 |  | Y |  |
| Site 1 | increasing oxygen requirement | 1 |  | Y |  |
| Site 1 | infection | 1 |  | Y |  |
| Site 1 | MISC | 1 | Y |  |  |
| Site 1 | myopathy | 4 |  | Y |  |
| Site 1 | nasuea | 1 |  | Y |  |
| Site 1 | nurologic and GI complaints | 1 |  | Y |  |
| Site 1 | nurological injury | 1 |  | Y |  |
| Site 1 | nuropathy | 1 |  | Y |  |
| Site 1 | PEA arrest | 3 | Y |  |  |
| Site 1 | PNA | 4 |  | Y |  |
| Site 1 | pneumothorax | 2 |  | Y |  |
| Site 1 | pulmonary infarction | 2 | Y |  |  |
| Site 1 | severe tricuspid regurgitation | 2 | Y |  |  |
| Site 1 | sinus yachycardia | 1 |  | Y |  |
| Site 1 | synopsizing | 1 |  | Y |  |
| Site 1 | systemic inflammatory response syndrome | 1 | Y |  |  |
| Site 1 | tachycardic | 2 |  | Y |  |
| Site 1 | temperature greater than 99.5 F | 1 |  | Y |  |
| Site 1 | thrombus | 3 |  | Y |  |
| Site 1 | vertiginous | 1 |  | Y |  |
| Site 1 | worsening of airspace | 1 | Y |  |  |
| Site 2 | CAP | 3 | Y |  |  |
| Site 2 | CHF | 1 | Y |  |  |
| Site 2 | OSA | 1 |  | Y |  |
| Site 3 | brain | 1 |  |  | Y |
| Site 3 | COVID | 2 |  |  | Y |
| Site 3 | COVID antibodies were positive | 1 |  | Y |  |
| Site 3 | COVID vaxxed | 1 |  | Y |  |
| Site 3 | COVID19 | 2 |  |  | Y |
| Site 3 | COVID-19 | 2 |  |  | Y |
| Site 3 | CTD | 1 |  | Y |  |
| Site 3 | CVA | 1 |  | Y |  |
| Site 3 | DM | 1 |  | Y |  |
| Site 3 | End Stage Renal Disease | 1 | Y |  |  |
| Site 3 | ESRD-End Stage Renal Disease | 1 | Y |  |  |
| Site 3 | h/o of covid | 1 |  | Y |  |
| Site 3 | High blood pressure: Positive: Mother | 1 |  | Y |  |
| Site 3 | HLD | 2 | Y |  |  |
| Site 3 | Hopeless | 1 |  | Y |  |
| Site 3 | HTN | 2 |  | Y |  |
| Site 3 | Hyperlipidemia | 1 | Y |  |  |
| Site 3 | ILD | 2 |  | Y |  |
| Site 3 | Increased body mass index | 1 | Y |  |  |
| Site 3 | Kidney transplant recipient | 1 | Y |  |  |
| Site 3 | mental processing is slower | 1 |  | Y |  |
| Site 3 | myopathy | 1 |  | Y |  |
| Site 3 | narcolepsy | 1 |  | Y |  |
| Site 3 | NKA | 1 | Y |  |  |
| Site 3 | Obesity | 1 | Y |  |  |
| Site 3 | OSA | 1 |  | Y |  |
| Site 3 | Periods are regular but heavy. | 1 |  | Y |  |
| Site 3 | Pfizer x2 | 1 |  |  | Y |
| Site 3 | PND | 1 |  | Y |  |
| Site 3 | post | 2 |  |  | Y |
| Site 3 | Post-acute sequelae of COVID-19 (PASC) | 1 | Y |  |  |
| Site 3 | Sleeping well | 1 |  |  | Y |
| Site 3 | w/ O2 88% | 1 |  | Y |  |
| Site 4 | Attention and concentration deficit | 1 |  | Y |  |
| Site 4 | attention deficit | 1 |  | Y |  |
| Site 4 | attention defictis | 1 |  | Y |  |
| Site 4 | cannot smell | 1 |  | Y |  |
| Site 4 | Cognitive Change | 1 |  | Y |  |
| Site 4 | could not remember things | 1 |  | Y |  |
| Site 4 | deconditioning | 5 | Y |  |  |
| Site 4 | difficulty getting good sleep | 1 |  | Y |  |
| Site 4 | difficulty with his taste | 1 |  | Y |  |
| Site 4 | difficulty with night sleep | 1 |  | Y |  |
| Site 4 | difficulty with thinking | 1 |  | Y |  |
| Site 4 | DOE | 1 |  | Y |  |
| Site 4 | HA | 3 |  | Y |  |
| Site 4 | imbalance | 2 |  | Y |  |
| Site 4 | lightheadedness | 1 |  | Y |  |
| Site 4 | Loss of awareness of body in space | 1 | Y |  |  |
| Site 4 | low energy | 1 |  | Y |  |
| Site 4 | menstrual cycles are off | 1 |  | Y |  |
| Site 4 | not been steady on his feet | 1 |  | Y |  |
| Site 4 | out of energy | 1 |  | Y |  |
| Site 4 | PND | 1 |  | Y |  |
| Site 4 | sinus tach | 1 |  | Y |  |
| Site 4 | sleep | 1 |  |  | Y |
| Site 4 | sleep has also been disrupted | 1 |  | Y |  |
| Site 4 | Sleep has been poor | 1 |  | Y |  |
| Site 4 | sleep wake reversal | 1 |  | Y |  |
| Site 4 | slept quite a bit | 1 |  | Y |  |
| Site 4 | steady on his feet | 1 |  |  | Y |
| Site 4 | taste | 1 |  |  | Y |
| Site 4 | text | 1 |  |  | Y |
| Site 4 | trauma | 1 | Y |  |  |
| Site 4 | unable to focus | 1 |  | Y |  |
| Site 4 | unable to function | 1 | Y |  |  |
| Site 4 | worsening of his taste | 1 |  | Y |  |
| Site 5 | COPD | 1 | Y |  |  |
| Site 5 | COVID | 33 |  |  | Y |
| Site 5 | COVID-19 | 5 |  |  | Y |
| Site 5 | forgetfullness | 1 |  | Y |  |
| Site 5 | GERD | 8 |  | Y |  |
| Site 5 | hearing decline | 1 |  | Y |  |
| Site 5 | long COVID | 10 |  |  | Y |
| Site 5 | POTS | 7 |  | Y |  |

“New concept” denotes that the clinical concept as a whole is not present within the dictionary. “New variant” denotes that the missed term is a new lexical variant for a concept that is present within the dictionary. Annotation error refers to annotations that fall outside of our task, for example, COVID-19 or long COVID is not a sign or symptom of COVID or post-acute sequelae of SARS CoV-2 infection (PASC).
